# Age-Related Patterns of Selected Protein-Altering Somatic Mutations and Overall Survival in Colorectal Cancer: A Retrospective Comparative Cohort Study

**DOI:** 10.3390/genes17101193

**Published:** 2026-09-26

**Authors:** Ege Rıza Karagür, Yasemin Adalı

**Affiliations:** 1 Department of Medical Genetics, Faculty of Medicine, Pamukkale University, 20070 Denizli, Türkiye; 2 Centre for Public Health, School of Medicine, Dentistry and Biomedical Sciences, Queen’s University Belfast, Belfast BT12 6BJ, UK; 3 Division of Epidemiology, Department of Public Health, Faculty of Medicine, Pamukkale University, 20070 Denizli, Türkiye

**Keywords:** early-onset colorectal cancer, colorectal cancer, somatic mutations, TCGA, clinicogenomics, overall survival, cancer epidemiology

## Abstract

**Background/Objectives**: Early-onset colorectal cancer (EOCRC) is increasing in incidence and may exhibit adverse clinicopathological features, but whether selected somatic mutations differ by age remains uncertain. We compared clinicopathological characteristics, protein-altering somatic mutations, and overall survival between EOCRC and late-onset colorectal cancer (LOCRC) using TCGA-COAD/READ data. **Methods**: We conducted a retrospective comparative cohort study using publicly available clinicogenomic data. Patients were classified as EOCRC (<50 years) or LOCRC (≥50 years). Mutation-prevalence analyses were restricted to patients with evaluable somatic mutation data and corrected for multiple testing. Overall survival and exploratory age-by-mutation interactions were assessed using Kaplan–Meier and Cox regression. **Results**: Among 627 patients with available age-at-diagnosis data, 77 had EOCRC and 550 had LOCRC. Somatic mutation data were evaluable for 74 and 504 patients, respectively. EOCRC was associated with higher AJCC pathological stage (*p* = 0.021) and greater pathological nodal involvement (*p* = 0.007). Stage IV disease was associated with higher adjusted odds of EOCRC (adjusted OR, 2.64; 95% CI, 1.10–6.71; *p* = 0.033). No selected mutation-prevalence comparison remained statistically significant after false discovery rate correction. *MSH2* mutation was nominally more frequent in EOCRC (8.1% vs. 2.8%; *p* = 0.032) but was nonsignificant after correction (q = 0.128). Age group was not associated with overall survival in the fully adjusted model (adjusted HR, 0.64; 95% CI, 0.33–1.25; *p* = 0.194). **Conclusions**: EOCRC was associated with higher pathological stage and greater nodal involvement in this TCGA cohort.

## 1. Introduction

Colorectal cancer (CRC) is a genetically heterogeneous malignancy that develops through the stepwise accumulation of somatic alterations affecting epithelial homeostasis, genome maintenance, and interactions with the tumor microenvironment [1,2]. In the conventional adenoma-carcinoma sequence, loss of *APC* function and subsequent activation of WNT/β-catenin signaling are typically early events. Additional changes involving the RAS-RAF-MAPK, PI3K-AKT, TP53, and TGF-β/SMAD pathways then support clonal expansion and malignant progression. CRC can also arise through other routes, most notably mismatch repair deficiency with microsatellite instability (MSI) and the serrated pathway, which is commonly associated with *BRAF* activation and CpG-island methylation. These pathways are not strictly separate, and their overlap contributes to the marked variation in tumor location, histology, immune phenotype, prognosis, and treatment response observed across CRCs [3,4]. Beyond these canonical pathways, less frequent but potentially actionable alterations involving receptor tyrosine kinases, including *ERBB2*/HER2, further contribute to the molecular heterogeneity and therapeutic stratification of colorectal cancer [5].

Early-onset colorectal cancer (EOCRC), usually defined as CRC diagnosed before 50 years of age, has become an increasingly important clinical problem. Incidence has risen in several populations even as rates among older adults have remained stable or declined, and younger patients are often diagnosed with regional or metastatic disease [6,7,8]. These trends further emphasize the need for improved early-detection strategies, including the investigation of circulating and other clinically accessible microRNAs as potential non-invasive biomarkers for colorectal cancer [9]. This pattern has prompted debate about the genetic basis of EOCRC. It remains unclear whether these tumors reflect a greater contribution from inherited susceptibility, a distinct form of sporadic CRC, or an earlier presentation of the same pathways that drive disease in older patients. Pathogenic germline variants in high-penetrance cancer-predisposition genes, particularly mismatch repair genes associated with Lynch syndrome and *APC* variants causing polyposis, explain a clinically important proportion of cases. However, most patients with EOCRC have no identifiable Mendelian disorder. Their tumors may instead arise from a combination of somatic evolution, polygenic susceptibility, epigenetic changes, environmental exposures, and host–microbiome interactions [10,11].

Studies comparing the somatic genomes of early- and later-onset tumors have not produced a consistent age-specific profile. *APC*, *TP53*, *KRAS*, *PIK3CA*, *SMAD4*, and *FBXW7* are among the most frequently altered genes in both groups [12,13,14,15]. A recent systematic review and meta-analysis reported lower pooled frequencies of *APC*, *KRAS*, and *BRAF* mutations, together with higher frequencies of *TP53* and *PTEN* mutations, in EOCRC [14]. Other cohort studies, particularly those restricted to microsatellite-stable or apparently sporadic tumors, found that the major driver alterations occurred at broadly similar frequencies across age groups [12,14,15]. Some of this disagreement probably reflects differences in sequencing platforms, age definitions, ancestry, tumor location, stage distribution, MSI status, exclusion of hereditary cases, and the denominators used to calculate mutation prevalence.

Somatic alterations should be interpreted within their broader signaling pathways rather than in isolation [16]. Somatic mutations in MMR genes alone do not establish MSI-H/dMMR status or Lynch syndrome and require complementary molecular testing and, where appropriate, germline evaluation.

It is also uncertain whether age changes the prognostic effect of recurrent driver mutations. Younger patients may present with more advanced disease, but they often have fewer competing causes of death and may receive more intensive treatment. Unadjusted survival comparisons can therefore be misleading because stage, tumor location, histological and molecular subtype, and treatment all influence outcome. *RAS* and *BRAF* alterations have established predictive or prognostic importance in specific clinical settings, whereas the reported survival effects of *APC*, *TP53*, *PIK3CA*, *SMAD4*, and *FBXW7* vary with disease context and co-mutation patterns [17]. Testing an age-by-mutation interaction is more appropriate than comparing whether a mutation is statistically significant in one age group but not the other. Such analyses, however, require sufficient mutation-positive events and correction for multiple testing.

Against this background, we used TCGA-COAD and TCGA-READ data to compare clinicopathological features and selected protein-altering somatic mutations between EOCRC and late-onset colorectal cancer (LOCRC). The analysis included selected genes from the WNT, TP53, RAS-RAF-MAPK, PI3K-AKT, TGF-β, ubiquitin-proteasome, and mismatch repair pathways. We also examined factors associated with EOCRC, compared overall survival after clinicopathological adjustment, and explored whether age group modified mutation-associated survival estimates. We expected EOCRC to be associated with higher pathological stage and greater pathological nodal involvement, while age-group differences in selected mutation prevalence were expected to be imprecisely estimated because of the limited EOCRC sample size.

## 2. Materials and Methods

### 2.1. Study Design and Data Source

This retrospective comparative cohort study was conducted using publicly available clinicogenomic data from The Cancer Genome Atlas Colon Adenocarcinoma (TCGA-COAD) and Rectum Adenocarcinoma (TCGA-READ) projects. Patients were grouped according to age at diagnosis and compared with respect to clinicopathological characteristics, selected protein-altering somatic mutations, and overall survival. Clinical and somatic mutation data were obtained from the National Cancer Institute Genomic Data Commons (GDC). Clinical information was retrieved separately for the TCGA-COAD and TCGA-READ projects and subsequently combined into a single colorectal cancer cohort [18]. Somatic mutation data were obtained from the GDC “Simple Nucleotide Variation” category using the “Masked Somatic Mutation” data type [18]. The reporting of this study followed the Strengthening the Reporting of Observational Studies in Epidemiology (STROBE) recommendations for cohort studies.

A total of 633 patients were initially identified, including 461 patients from TCGA-COAD and 172 from TCGA-READ. Age at diagnosis was unavailable for six patients; therefore, 627 patients were included in the age-stratified analyses. Patients diagnosed before 50 years of age were classified as having early-onset colorectal cancer (EOCRC), whereas those diagnosed at 50 years of age or older were classified as having late-onset colorectal cancer (LOCRC). The final age-defined cohort comprised 77 patients with EOCRC and 550 patients with LOCRC.

### 2.2. Clinical and Clinicopathological Variables

Clinical variables extracted from the GDC included age at diagnosis, sex, year of diagnosis, TCGA project, tumor location, histological subtype, AJCC pathological stage, pathological T, N, and M categories, prior malignancy, synchronous malignancy, residual disease, vital status, days to death, and days to last follow-up.

Age at diagnosis was calculated from the reported age in days and expressed in years. Overall survival time was defined as the interval from diagnosis to death for deceased patients or to the last available follow-up for patients who were alive.

Tumor location was categorized as right colon, left colon, rectosigmoid junction, or rectum. Right-sided tumors included tumors of the cecum, ascending colon, hepatic flexure, and transverse colon, whereas left-sided tumors included tumors of the splenic flexure, descending colon, and sigmoid colon. Rectosigmoid and rectal tumors were analyzed as separate anatomical categories.

AJCC pathological stage subcategories were consolidated into Stages I–IV. Pathological T categories were grouped as T1–T4, N categories as N0–N2, and M categories as M0–M1. R1 and R2 residual disease categories were combined, with R0 used as the reference category. “Not reported” values for prior malignancy and synchronous malignancy were treated as missing and excluded from the corresponding binary analyses. These classifications were applied using an available-case approach because the completeness of clinicopathological variables differed across patients.

Detailed treatment variables were not included in the regression models because information on systemic treatment regimens, treatment intensity, dose, timing, adherence, and subsequent lines of therapy was incomplete and not consistently harmonized across TCGA-COAD and TCGA-READ.

### 2.3. Somatic Mutation Data Processing

Somatic mutation data were processed at the patient level using tumor sample barcodes truncated to the first 12 characters to obtain the corresponding TCGA patient identifier. The analysis focused on a predefined set of colorectal cancer-related genes: *APC*, *TP53*, *KRAS*, *NRAS*, *BRAF*, *PIK3CA*, *SMAD4*, *FBXW7*, *TGFBR2*, *MLH1*, *MSH2*, *MSH6*, and *PMS2*.

The GDC data were accessed on 9 September 2026. Masked somatic mutation files generated using the GDC Aliquot Ensemble Somatic Variant Merging and Masking workflow were queried for TCGA-COAD and TCGA-READ. Mutation evaluability was established independently from the GDC file metadata and verified against the patients represented in the mutation file. Of the 627 patients with known age at diagnosis, 578 had evaluable somatic mutation data, including 74 with EOCRC and 504 with LOCRC. Patients without evaluable mutation data were assigned missing mutation status and excluded from all mutation-prevalence and mutation-associated regression analyses; they were not classified as wild type. Multiple tumor aliquots from the same patient were collapsed to a single patient-level identifier.

To restrict the analysis to potentially protein-altering events, only the following somatic variant classifications were retained: missense mutations, nonsense mutations, frame-shift deletions, frame-shift insertions, in-frame deletions, in-frame insertions, splice-site variants, and splice-region variants. Silent mutations, untranslated-region variants, intronic variants, and other non-protein-altering classifications were excluded.

For each gene, mutation status was coded at the patient level as mutant if at least one qualifying protein-altering somatic variant was present and as wild type otherwise. A composite *RAS* mutation variable was defined as the presence of a *KRAS* or *NRAS* mutation. A composite MMR-gene mutation variable was defined as a qualifying somatic mutation in at least one of *MLH1*, *MSH2*, *MSH6*, or *PMS2*. The latter was used strictly as a somatic mutation composite and was not interpreted as equivalent to microsatellite instability-high (MSI-H) or deficient mismatch repair (dMMR) status. To explore variant-level heterogeneity that could not be captured by the primary gene-level classification, additional descriptive analyses were performed for *KRAS*, *BRAF*, and *PIK3CA*. *KRAS* alterations were grouped according to codons 12, 13, 61, 117, and 146, with the remaining alterations classified as other *KRAS* variants. *BRAF* alterations were categorized as V600E hotspot-equivalent or non-V600E variants. The GDC transcript annotation used in this dataset labels the canonical *BRAF* hotspot-equivalent event as p.V640E; therefore, hotspot-equivalent terminology was used without assuming canonical transcript equivalence. *PIK3CA* alterations were grouped into the helical-domain hotspot region, kinase-domain hotspot region, and other *PIK3CA* variants. Because some patients carried more than one qualifying alteration, variant-level categories were not necessarily mutually exclusive. Somatic mutation data processing and visualization were performed using the maftools package 2.26.0 [19].

Functional MSI status was obtained from GDC whole-exome sequencing aligned-read metadata generated using MSIsensor2, reported as version 0.1 in the software repository. Primary-tumor records with nonmissing MSI status were retained. Patients with discordant MSI and MSS classifications across available records were excluded from MSI-based analyses. MSI status was available and concordant for 74 EOCRC and 514 LOCRC patients; MSI-adjusted analyses additionally required evaluable somatic mutation data.

### 2.4. Outcome Definition

The primary survival outcome was overall survival (OS). Survival time was calculated using days to death for deceased patients and days to last follow-up for patients who were alive. Survival time was converted from days to months using 30.44 days per month. Overall survival was the only time-to-event endpoint evaluated. The available clinical data did not support classification of cause-specific mortality or consistent derivation of disease-free survival. Therefore, these outcomes were not analyzed.

For the primary survival analysis, patients with a recorded survival time of zero days were retained by assigning a minimal survival duration of 0.5 days to permit inclusion in time-to-event analyses. Patients without sufficient survival information were excluded. The resulting primary survival cohort included 625 patients, with 129 deaths. In the fully adjusted clinicopathological Cox model, complete-case availability of covariates reduced the analytical sample to 584 patients with 114 deaths.

### 2.5. Statistical Analysis

Continuous variables were summarized using medians and interquartile ranges (IQRs), whereas categorical variables were presented as frequencies and percentages. Comparisons between EOCRC and LOCRC were performed using Pearson’s chi-square test for categorical variables. Fisher’s exact test was used when expected cell counts were sparse. Year of diagnosis was compared using the Wilcoxon rank-sum test. No statistical comparison was performed for age at diagnosis because age was used to define the study groups.

Associations between individual somatic mutations and EOCRC status were evaluated using logistic regression, with LOCRC serving as the reference group. Odds ratios (ORs) and 95% confidence intervals (CIs) were reported. Individual MMR-gene mutation comparisons are presented in Supplementary Table S1.

Factors associated with EOCRC were further assessed using univariable and multivariable logistic regression models. The exploratory multivariable model included diagnosis period, sex, AJCC pathological stage, prior malignancy, and *FBXW7* mutation status. *FBXW7* was included as an exploratory genomic covariate because it showed the largest numerical difference in mutation prevalence between the age groups among the selected recurrent genes, although its univariable association was not statistically significant. This exploratory modeling choice was not based on statistical significance and did not constitute an automated variable-selection procedure. Diagnosis period was categorized as 1998–2005, 2006–2009, and 2010–2013. Sensitivity analyses were performed by replacing AJCC pathological stage with pathological N category, expanding the model to include tumor location and mucinous histology, and repeating the primary model without prior malignancy because only one EOCRC patient had a documented prior malignancy. The model excluding prior malignancy otherwise retained diagnosis period, sex, AJCC pathological stage, and *FBXW7* mutation status. The nodal sensitivity model and the expanded model including tumor location and mucinous histology are presented in Supplementary Tables S2 and S3, respectively.

Overall survival was analyzed using Kaplan–Meier methods and Cox proportional hazards regression. The primary Cox models were constructed sequentially as follows: Model 1 included age group alone; Model 2 additionally adjusted for sex and diagnosis period; and Model 3 additionally adjusted for AJCC pathological stage, tumor location, and mucinous histology. Hazard ratios (HRs) with 95% CIs were reported. The proportional hazards assumption was assessed using Schoenfeld residual-based tests [20].

To investigate whether the associations between somatic mutation status and overall survival differed by age group, age group-by-mutation interaction terms were evaluated in Cox proportional hazards models for *APC*, *TP53*, *RAS*, *BRAF*, *PIK3CA*, *SMAD4*, *FBXW7*, and the MMR-gene composite. These analyses were restricted to patients with evaluable somatic mutation data and available overall survival information. Mutation-specific hazard ratios were estimated separately within the EOCRC and LOCRC groups, and the corresponding interaction *p* values were adjusted for multiple comparisons using the Benjamini–Hochberg false discovery rate (FDR) method [21]. Benjamini–Hochberg FDR-adjusted q values were also calculated across the 11 prespecified mutation-prevalence comparisons. To assess potential confounding by functional MSI status, the associations between individual mutations and EOCRC were re-estimated using logistic regression models adjusted for patient-level MSI status. Mutation-prevalence comparisons were also repeated after restriction to patients with microsatellite-stable tumors. Patients with discordant MSI classifications across available tumor records were excluded from the MSI-based sensitivity analyses. The fully adjusted age-by-BRAF interaction analysis is presented in Supplementary Table S4.

Additional sensitivity analyses included exclusion of patients with zero-day survival, application of an alternative EOCRC cutoff of <45 years, and restriction of the survival analysis to the TCGA-COAD cohort. All statistical tests were two-sided. *p* values < 0.05 were considered statistically significant, and for analyses subject to multiple-testing correction, statistical significance was assessed using an FDR-adjusted q value < 0.05. Clinical statistical analyses were performed in Stata/IC version 16.1 (StataCorp, College Station, TX, USA), and the logistic and survival models were additionally reproduced or verified in R. Data acquisition, mutation processing, MSI-based analyses, statistical verification, and graphical analyses were performed in R version 4.5.2 (R Foundation for Statistical Computing, Vienna, Austria) using TCGAbiolinks version 2.38.0 [22], maftools version 2.26.0 [19], survival version 3.8-3, broom version 1.0.11, dplyr version 1.1.4, tidyr version 1.3.2, stringr version 1.6.0, and purrr version 1.2.1. The complete R script is provided as Supplementary File S1. The corresponding analyses excluding patients with zero-day survival, applying an EOCRC cutoff of <45 years, and restricting the analysis to TCGA-COAD are presented in Supplementary Tables S5–S7, respectively.

### 2.6. Ethical Considerations

This study exclusively involved the secondary analysis of publicly available, de-identified data and metadata obtained from the NCI Genomic Data Commons. The investigators had no interaction with participants, collected no new biological specimens or clinical information, and did not access identifiable or controlled-access individual-level data. The original collection of TCGA biospecimens and clinical data was conducted by the participating institutions under their respective institutional review processes and informed-consent requirements. Therefore, additional approval from the authors’ institutional ethics committee was not required for the present secondary analysis.

## 3. Results

### 3.1. Study Cohort and Analytical Framework

The combined TCGA-COAD/READ cohort included 633 patients, of whom 461 were from TCGA-COAD and 172 from TCGA-READ. Age at diagnosis was unavailable for six patients, leaving 627 patients for age-stratified analyses. Of these, 77 (12.3%) were classified as EOCRC and 550 (87.7%) as LOCRC. The overall-survival analysis included 625 patients with available survival time and event status. The cohort selection and analytical framework are summarized in Figure 1. The clinicopathological characteristics of the age-defined cohort are presented in Table 1.

Somatic mutation data were evaluable for 578 of the 627 age-defined patients: 74/77 patients with EOCRC (96.1%) and 504/550 patients with LOCRC (91.6%). The remaining 3 EOCRC and 46 LOCRC patients were excluded from mutation-based analyses and were not classified as wild type (Supplementary Table S8). Mutation-based survival analyses included 577 patients with both evaluable mutation and overall-survival data (74 EOCRC and 503 LOCRC; 125 deaths, including 10 EOCRC deaths).

### 3.2. Selected Protein-Altering Somatic Mutation Profiles

Within the EOCRC group, the selected protein-altering somatic mutations most frequently involved *APC*, *TP53*, *KRAS*, *PIK3CA*, *FBXW7*, *SMAD4*, and *BRAF*, whereas alterations in the individual MMR genes, *TGFBR2*, and *NRAS* were less frequent. Figure 2 displays the distribution and variant classes of the selected protein-altering somatic mutations among EOCRC tumors represented in the mutation dataset.

In LOCRC, *APC*, *TP53*, *KRAS*, and *PIK3CA* were also among the most frequently altered genes within the predefined gene set. The larger LOCRC cohort contained greater absolute numbers of mutation-positive tumors (Figure 3).

Mutation-prevalence analyses were restricted to the 578 patients with evaluable somatic mutation data (74 EOCRC and 504 LOCRC). The 3 EOCRC and 46 LOCRC patients without evaluable mutation data were excluded and were not classified as wild type. *APC* mutations were detected in 48/74 EOCRC patients (64.9%) and 380/504 LOCRC patients (75.4%); TP53 mutations in 44/74 (59.5%) and 300/504 (59.5%); and *RAS* mutations in 29/74 (39.2%) and 247/504 (49.0%), respectively. No comparison remained statistically significant after Benjamini–Hochberg false discovery rate correction (Table 2 and Figure 4).

### 3.3. Clinicopathological Characteristics According to Age at Diagnosis

The median age at diagnosis was 45.2 years (IQR, 40.7–47.4) in EOCRC and 69.9 years (IQR, 62.4–77.8) in LOCRC. EOCRC patients were diagnosed slightly later in calendar time (median 2010 vs. 2009; *p* = 0.007). The distribution of AJCC pathological stage differed significantly by age group (*p* = 0.021), with Stage III and IV disease accounting for 61.3% of EOCRC compared with 42.3% of LOCRC. Pathological nodal status also differed significantly (*p* = 0.007), with N1/N2 disease observed in 59.7% of EOCRC versus 40.8% of LOCRC. Prior malignancy was less frequent among EOCRC patients (1.3% vs. 12.4%; *p* = 0.001). Other evaluated clinicopathological characteristics did not show statistically significant age-group differences (Table 1).

### 3.4. Age-Related Patterns of Selected Protein-Altering Somatic Mutations

Among mutation-evaluable patients, no selected individual or composite mutation variable differed statistically between EOCRC and LOCRC after false discovery rate correction. *FBXW7* mutations occurred in 15/74 EOCRC patients (20.3%) and 79/504 LOCRC patients (15.7%; OR, 1.37; 95% CI, 0.68–2.59; *p* = 0.314; q = 0.691). *APC* and *RAS* mutations were numerically less frequent in EOCRC, but their confidence intervals remained compatible with potentially meaningful differences. *BRAF* mutations occurred in 8/74 EOCRC patients (10.8%) and 58/504 LOCRC patients (11.5%; *p* = 1.000; q = 1.000) (Table 2). A descriptive variant-level analysis demonstrated heterogeneity within *KRAS*, *BRAF*, and *PIK3CA* alterations (Supplementary Table S9). *KRAS* codon 12 alterations were the predominant *KRAS* subgroup in both groups. *BRAF* hotspot-equivalent alterations were observed in 3 EOCRC and 47 LOCRC patients, whereas non-V600E *BRAF* alterations occurred in 5 and 15 patients, respectively. *PIK3CA* helical-domain hotspot-region alterations occurred in 5 EOCRC and 59 LOCRC patients, and kinase-domain hotspot-region alterations occurred in 3 and 34 patients, respectively. Because individual EOCRC variant subgroups were small and categories were not necessarily mutually exclusive, these results were treated as descriptive.

### 3.5. Factors Associated with Early-Onset Colorectal Cancer

In univariable analyses, Stage III and Stage IV disease each showed approximately two-fold higher odds of EOCRC relative to Stage I. In the multivariable model (*n* = 555; 72 EOCRC and 483 LOCRC), Stage IV disease was associated with higher odds of EOCRC relative to Stage I (adjusted OR, 2.64; 95% CI, 1.10–6.71; *p* = 0.033). A history of prior malignancy was inversely associated with EOCRC (adjusted OR, 0.12; 95% CI, 0.01–0.56; *p* = 0.037), although this estimate should be interpreted cautiously because only one EOCRC patient had a documented prior malignancy. *FBXW7* mutation status was not associated with EOCRC after multivariable adjustment (adjusted OR, 1.46; 95% CI, 0.75–2.73; *p* = 0.250) (Table 3). When prior malignancy was omitted because of the single exposed EOCRC patient, Stage IV pathological disease remained associated with higher odds of EOCRC (adjusted OR, 2.49; 95% CI, 1.04–6.29; *p* = 0.044), whereas *FBXW7* mutation remained statistically nonsignificant (adjusted OR, 1.47; 95% CI, 0.75–2.73; *p* = 0.240).

### 3.6. Overall Survival

Age group was not significantly associated with overall survival in the unadjusted model (HR, 0.60; 95% CI, 0.31–1.14; *p* = 0.116) or after adjustment for sex and diagnosis period (HR, 0.64; 95% CI, 0.34–1.24; *p* = 0.186). In the fully adjusted clinicopathological model, EOCRC remained unassociated with overall survival (adjusted HR, 0.64; 95% CI, 0.33–1.25; *p* = 0.194). In contrast, pathological stage was strongly associated with mortality: compared with Stage I, Stage III and Stage IV disease was associated with adjusted HRs of 3.69 (*p* = 0.003) and 10.09 (*p* < 0.001), respectively. Left-colon and rectal tumor locations were associated with lower hazards relative to right-colon tumors. The global proportional hazards test for the fully adjusted model was not statistically significant (*p* = 0.091) (Table 4).

### 3.7. Age-Specific Prognostic Associations of Somatic Mutations

No age-by-mutation interaction remained statistically significant after FDR correction. The largest point estimate was observed for *BRAF*. *BRAF* mutation was not associated with overall survival in LOCRC (HR, 1.01; 95% CI, 0.57–1.80), whereas the corresponding estimate in EOCRC was HR 4.37 (95% CI, 0.92–20.74). However, only two deaths occurred among the eight *BRAF*-mutant EOCRC patients, and the age-by-*BRAF* interaction was not statistically significant (interaction HR, 4.33; 95% CI, 0.82–22.82; *p* = 0.084; FDR q = 0.670). In the fully adjusted model, diagnosis period and tumor location violated the proportional-hazards assumption; a model stratified by these variables satisfied the global assumption (*p* = 0.170). In that stratified model, the age-by-*BRAF* interaction remained nonsignificant (interaction HR, 4.00; 95% CI, 0.71–22.45; *p* = 0.115). The age-specific estimates and interaction results for all examined mutations are presented in Table 5.

Functional MSI status did not differ statistically between age groups: MSI was identified in 9/74 EOCRC patients (12.2%) and 71/514 LOCRC patients (13.8%). Five patients with discordant MSI/MSS records were excluded from MSI-based sensitivity analyses. In MSI-adjusted models including 572 patients with complete MSI and mutation data (73 EOCRC and 499 LOCRC), no selected mutation association remained significant after FDR correction. Results were likewise nonsignificant after correction in the MSS-restricted analysis of 495 mutation-evaluable patients (64 EOCRC and 431 LOCRC) (Supplementary Tables S10–S12). Detailed patient and death counts for the exploratory age-by-mutation analyses are provided in Supplementary Table S13, and the sensitivity model excluding prior malignancy is presented in Supplementary Table S14.

Exploratory and sensitivity analyses are provided in the Supplementary Materials. Individual MMR-gene analysis showed a nominally higher *MSH2* mutation frequency in EOCRC (8.1% vs. 2.8%; *p* = 0.032), but this association did not remain significant after FDR correction (q = 0.128). Sensitivity models replacing pathological stage with nodal category, excluding prior malignancy from the EOCRC model, excluding zero-day survival observations, applying an EOCRC cutoff of <45 years, restricting survival analyses to TCGA-COAD, adjusting mutation comparisons for MSI, and restricting mutation comparisons to MSS tumors were consistent with the primary interpretation.

## 4. Discussion

In this TCGA cohort, EOCRC was associated with higher pathological stage and greater pathological nodal involvement. None of the selected protein-altering somatic mutation comparisons remained statistically significant after false discovery rate correction, although the confidence intervals did not exclude potentially meaningful age-group differences. *APC*, *TP53*, and RAS-pathway mutations were the most frequently detected alterations among the selected genes in both groups. Age group was not statistically associated with overall survival in the fully adjusted model, whereas pathological stage remained strongly associated with mortality. These cohort-specific associations do not establish molecular equivalence or show that the observed pathological differences are caused independently by age or individual mutations.

Although no selected mutation comparison remained statistically significant after FDR correction, colorectal tumors arising at different ages may still converge on shared core oncogenic pathways [1,3,4]. This interpretation is also compatible with large genomic series showing that many recurrent driver alterations are shared between early- and later-onset disease [14,15,23]. They differ from pooled analyses reporting modest reductions in *APC*, *KRAS*, and *BRAF* mutations and enrichment of *TP53* or *PTEN* mutations in EOCRC [13]. This inconsistency is not unexpected. Age thresholds, tumor location, MSI composition, ancestry, stage distribution, sequencing platforms, and the handling of hereditary cases vary considerably between studies and can influence apparently age-related mutation frequencies.

*APC* mutations were detected in 64.9% of mutation-evaluable EOCRC patients and 75.4% of mutation-evaluable LOCRC patients. Although the point estimate suggested a lower prevalence in younger patients, the comparison was not statistically significant after false discovery rate correction (OR, 0.60; 95% CI, 0.35–1.06; *p* = 0.064; q = 0.691). The confidence interval does not exclude a potentially meaningful difference. Moreover, absence of a qualifying *APC* sequence variant does not imply intact WNT signaling because pathway activity may also be influenced by copy-number changes, loss of heterozygosity, structural alterations, or alterations in other WNT-pathway components. Therefore, these data provide insufficient evidence of an age-group difference in selected *APC* coding mutations and do not establish equivalent WNT-pathway activity.

*TP53* mutations were detected in 59.5% of mutation-evaluable EOCRC patients and 59.5% of mutation-evaluable LOCRC patients (OR, 1.00; 95% CI, 0.59–1.70; *p* = 1.000; q = 1.000). This analysis detected no statistically significant age-group difference, but its precision does not demonstrate equivalence. Truncating variants, DNA-binding-domain missense variants, dominant-negative alleles, and gain-of-function variants may have different biological consequences. In addition, *TP53* status is shaped by 17p loss, copy-neutral loss of heterozygosity, aneuploidy, and whole-genome doubling, none of which was captured by the patient-level mutation indicator.

No statistically significant age-group differences were detected for *KRAS*, *NRAS*, composite *RAS*, *BRAF*, or *PIK3CA* mutations after false discovery rate correction. Composite *RAS* mutations occurred in 39.2% of mutation-evaluable EOCRC patients and 49.0% of mutation-evaluable LOCRC patients (OR, 0.67; 95% CI, 0.39–1.13; *p* = 0.135; q = 0.691), whereas *BRAF* mutations occurred in 10.8% and 11.5%, respectively. These nonsignificant comparisons should not be interpreted as demonstrating molecular equivalence. Gene-level indicators may obscure clinically and biologically relevant variation among *KRAS* codons, *BRAF* hotspot and non-hotspot alterations, and *PIK3CA* domains. Therefore, the absence of statistically significant gene-level differences does not establish equivalent pathway biology across age groups.

*FBXW7* mutation frequency was numerically higher in mutation-evaluable EOCRC than LOCRC (20.3% versus 15.7%), but the association was not statistically significant in the prevalence analysis (OR, 1.37; 95% CI, 0.68–2.59; *p* = 0.314; q = 0.691) or after multivariable adjustment. Accordingly, the data provide insufficient evidence that *FBXW7* mutation is an age-associated genomic feature. The variable was retained in the exploratory multivariable EOCRC model because it showed the largest numerical difference among the prespecified recurrent genes, rather than because of statistical significance. Larger studies with broader pathway-level profiling are required to estimate this association more precisely [24].

The mismatch repair findings require restraint. *MSH2* mutations were nominally more frequent in EOCRC (8.1% versus 2.8%; *p* = 0.032), but the association was not significant after FDR correction (q = 0.128). Functional MSI status was therefore evaluated separately. MSI prevalence did not differ statistically by age group, and neither MSI-adjusted nor MSS-restricted sensitivity analyses identified an association that remained significant after FDR correction. A somatic coding mutation in *MLH1*, *MSH2*, *MSH6*, or *PMS2* is not equivalent to functional dMMR, MSI-high status, or Lynch syndrome. Mismatch repair loss may involve biallelic sequence alterations, loss of heterozygosity, or *MLH1* promoter methylation, whereas Lynch syndrome requires germline evaluation. The present MMR-gene composite consequently describes only selected somatic coding mutations and cannot determine the functional or hereditary origin of mismatch repair deficiency [25,26,27].

The coexistence of higher pathological stage and greater pathological nodal involvement with no statistically significant differences in the selected mutation comparisons suggests that relevant age-related features may lie outside the alterations evaluated here. Diagnostic delay in younger adults was not measured in the present dataset and therefore could not be evaluated as a potential contributor. Somatic copy-number alterations, structural variants, non-coding regulatory changes, DNA methylation, chromatin state, transcriptional programs, immune composition, and microbiome-associated mutational processes were not examined [28,29]. Tumors harboring alterations in the same driver genes may nevertheless differ in mutation-acquisition order, clonality, subclonality, and co-occurrence patterns [30]. These molecular and evolutionary dimensions may be more informative than individual gene-level prevalence comparisons when investigating age-related differences in tumor biology.

Age group was not significantly associated with overall survival in the unadjusted or adjusted models. In the fully adjusted analysis, the hazard ratio for EOCRC was 0.64 (95% CI, 0.33–1.25; *p* = 0.194), whereas Stage III and Stage IV disease was associated with substantially higher mortality. Younger patients may have fewer competing causes of death and may receive more intensive therapy, but treatment data were not available for adequate adjustment. These findings indicate that age group was not statistically associated with OS after adjustment for the available clinicopathological covariates, whereas pathological stage remained associated with mortality.

Previous studies have generally associated *BRAF* mutation, particularly *BRAF* V600E, with adverse outcomes in colorectal cancer. Phipps et al. reported poorer disease-specific survival among patients with *BRAF*-mutant tumors after adjustment for stage, MSI status, and other patient and tumor characteristics [31]. However, evidence regarding whether this prognostic association differs according to age remains limited. In the CALGB/SWOG 80405 metastatic colorectal cancer cohort, overall survival did not differ significantly between younger and older patients, whereas younger age was associated with better overall survival within the *BRAF*-mutant subgroup [32]. This finding differs directionally from the exploratory point estimate observed in the present study. However, the CALGB/SWOG 80405 analysis included a uniformly treated metastatic population and evaluated the association of age within *BRAF*-defined subgroups, whereas the present analysis included patients across different pathological stages and estimated the prognostic association of *BRAF* mutation within each age group. In addition, the present study classified all qualifying protein-altering *BRAF* variants at the gene level rather than restricting the analysis to *BRAF* V600E. The findings are therefore not directly comparable. Moreover, the *BRAF* estimate in EOCRC was based on only eight mutation-positive patients and two deaths, resulting in a wide confidence interval. It should consequently be interpreted as exploratory and hypothesis-generating.

This study has several limitations. First, TCGA is not a population-based cohort, and selection related to referral patterns, tissue availability, ancestry, stage distribution, and follow-up may limit generalizability. Second, only 77 patients met the EOCRC definition, with 10 deaths. Several mutation-defined subgroups therefore contained very few events; notably, only two deaths occurred among eight patients with *BRAF*-mutant EOCRC. The resulting wide confidence intervals limit the precision and power of the age-by-mutation analyses, which should be considered exploratory and hypothesis-generating. Third, the primary analyses classified selected protein-altering variants at the gene level and did not capture variant-specific oncogenicity, copy-number alterations, loss of heterozygosity, structural variation, or clonal architecture. Although descriptive codon-, hotspot-, and domain-level analyses were performed for *KRAS*, *BRAF*, and *PIK3CA*, the small subgroup sizes precluded reliable inferential comparisons. Fourth, MSI status was derived from available GDC whole-exome sequencing metadata and was unavailable or discordant for some patients; *MLH1* methylation, germline predisposition, and other mechanisms underlying mismatch repair deficiency were not evaluated. Cause-specific mortality and disease-free survival could not be assessed because the available clinical data did not support consistent classification of cause of death or recurrence. Fifth, harmonized information on comorbidities, performance status, surgical procedures, and systemic treatment was unavailable. Residual confounding by these factors may have influenced the observed clinical and survival associations, particularly because treatment intensity may differ according to age. Accordingly, the absence of a statistically significant survival difference should not be interpreted as evidence of equivalent prognosis or treatment response. Finally, staging was based primarily on pathological rather than clinical stage and may not fully reflect disease extent at initial presentation. Thus, the findings describe cohort-specific associations involving selected somatic coding mutations and clinicopathological characteristics rather than the complete molecular or clinical distinction between EOCRC and LOCRC.

The primary mutant-versus-wild-type classification may obscure biologically relevant codon-, domain-, and variant-specific differences. Although descriptive analyses demonstrated heterogeneity within *KRAS*, *BRAF*, and *PIK3CA* alterations, the small numbers of EOCRC patients within individual variant subgroups precluded adequately powered inferential comparisons. Future studies should integrate germline susceptibility with MSI/MMR status, copy-number and structural alterations, epigenetic and transcriptional profiles, variant-level oncogenicity, and clonality. Validation in larger, ancestry-diverse cohorts with harmonized treatment and outcome data is needed, with hereditary, MSI-high/dMMR, and microsatellite-stable sporadic tumors evaluated separately where possible.

In the TCGA-COAD/READ cohort, EOCRC was associated with higher pathological stage and greater pathological nodal involvement. No statistically significant age-group differences were detected among the selected protein-altering somatic mutations after false discovery rate correction. These cohort-specific findings do not establish molecular equivalence or demonstrate that the observed pathological differences are independently attributable to age or individual mutations.

## 5. Conclusions

In this TCGA-COAD/READ cohort, EOCRC was associated with higher pathological stage and greater pathological nodal involvement than LOCRC. No statistically significant differences in the selected protein-altering somatic mutations were detected between the age groups after FDR correction. Age group was not statistically associated with overall survival in the adjusted analysis. However, the limited number of EOCRC patients and deaths, the inability to evaluate cause-specific mortality and disease-free survival, and the absence of harmonized information on comorbidities, surgical procedures, and systemic treatment preclude conclusions regarding equivalent prognosis or independent biological effects of age and individual somatic mutations. These findings should therefore be interpreted cautiously as cohort-specific associations that require validation in larger datasets with more comprehensive clinical and treatment information.

## Figures and Tables

**Figure 1:**
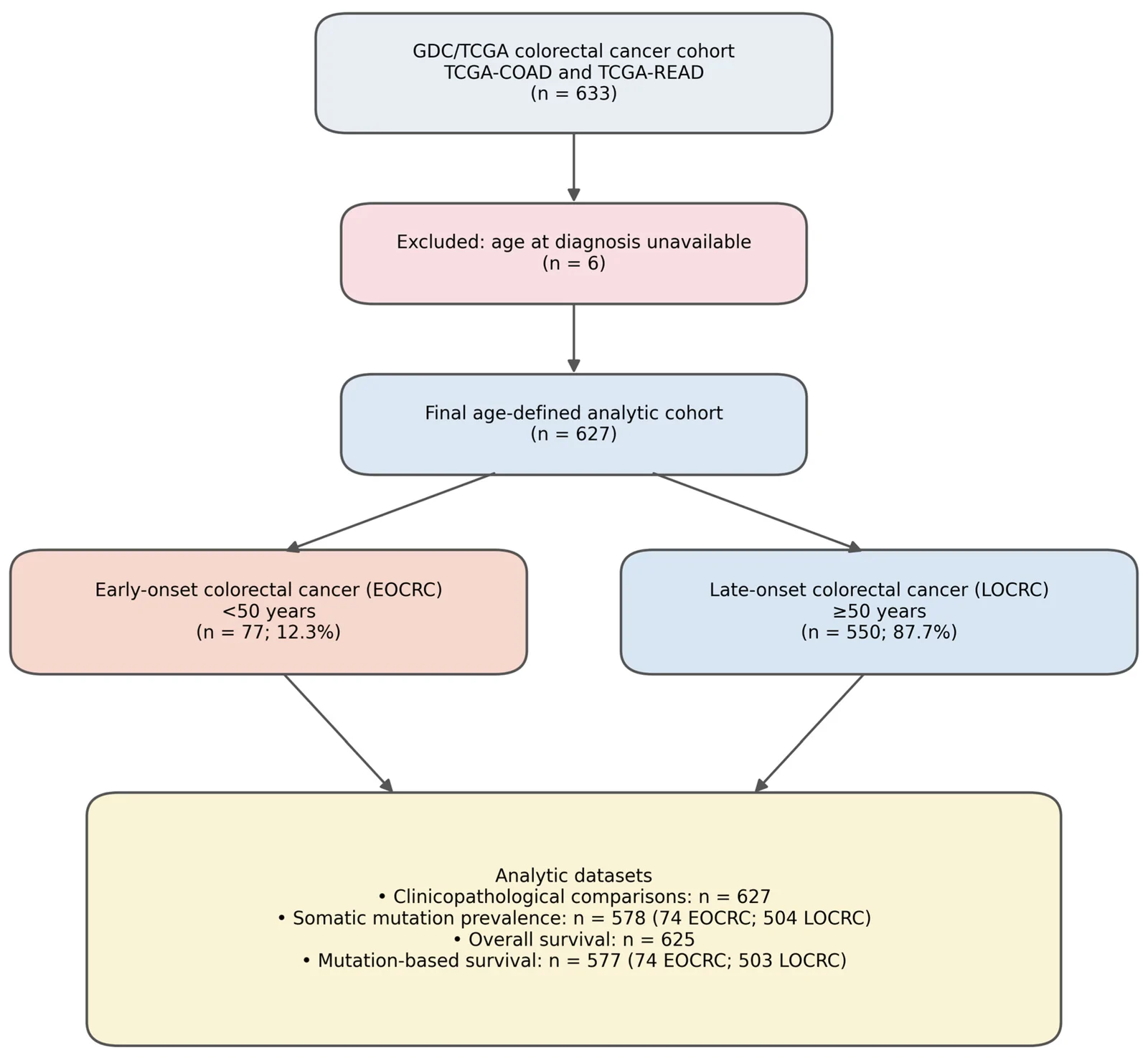
Flow diagram of patient selection and the analytical framework for the TCGA-COAD/READ colorectal cancer cohort.

**Figure 2:**
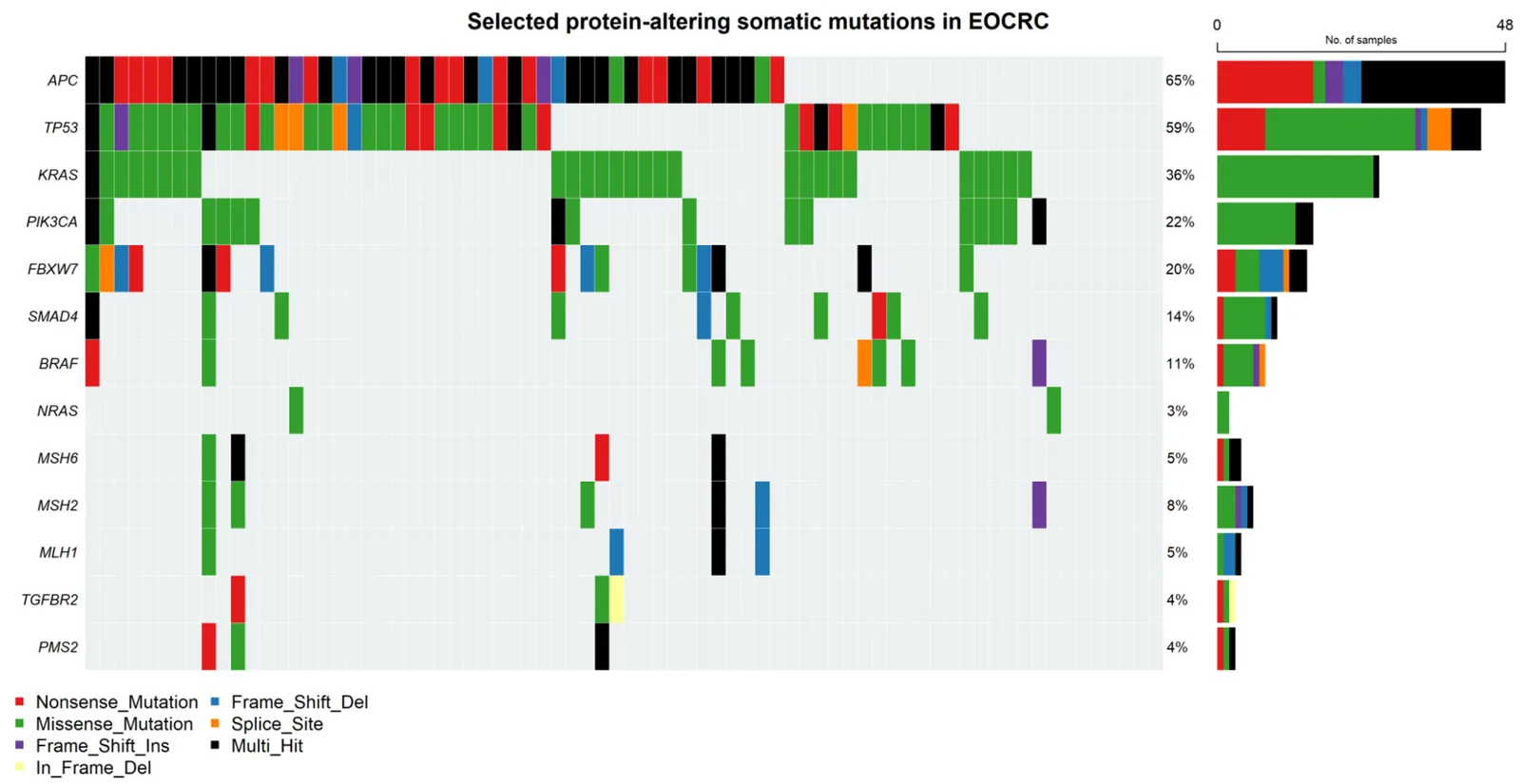
Selected protein-altering somatic mutations in early-onset colorectal cancer among 74 mutation-evaluable patients.

**Figure 3:**
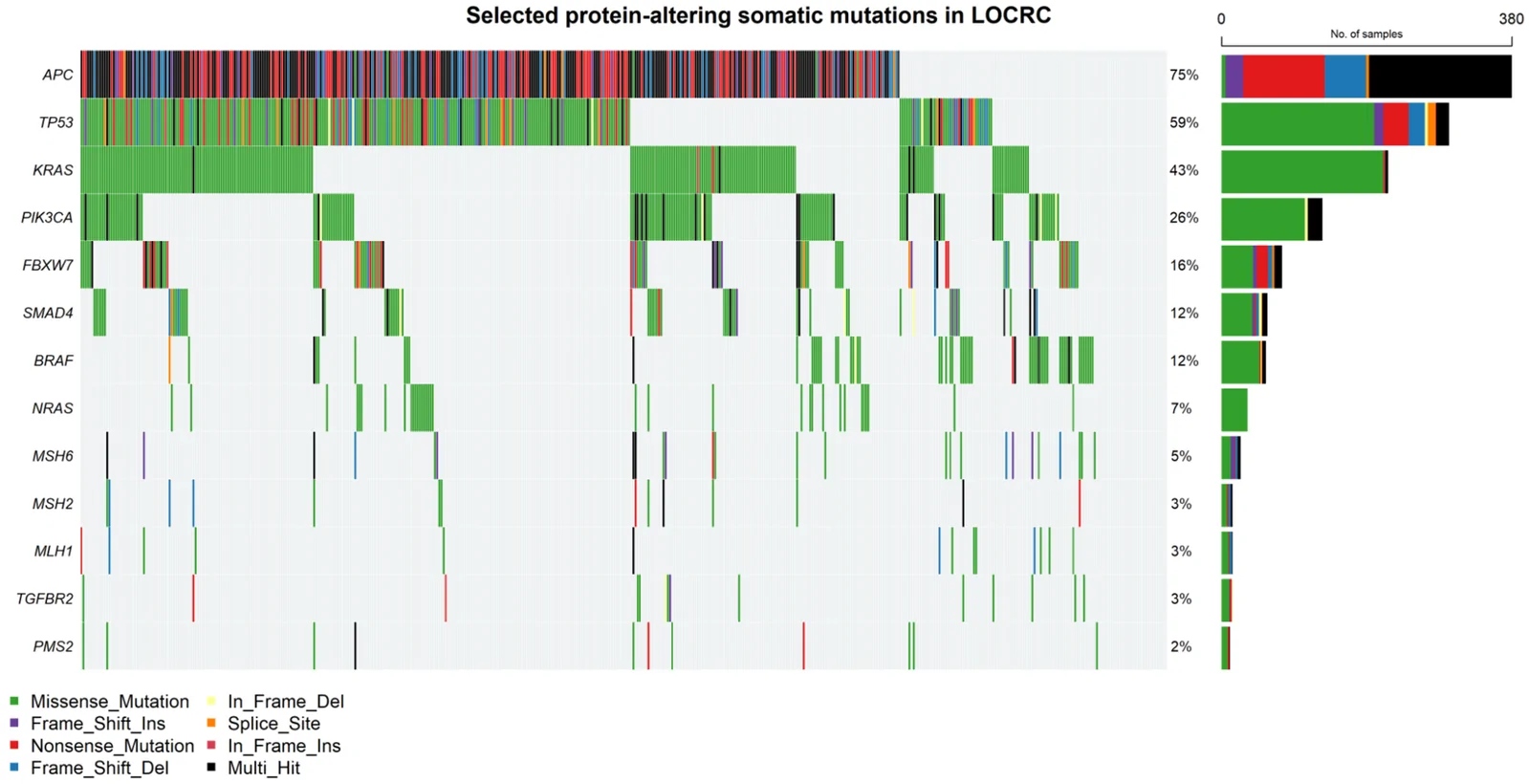
Selected protein-altering somatic mutations in late-onset colorectal cancer among 504 mutation-evaluable patients.

**Figure 4:**
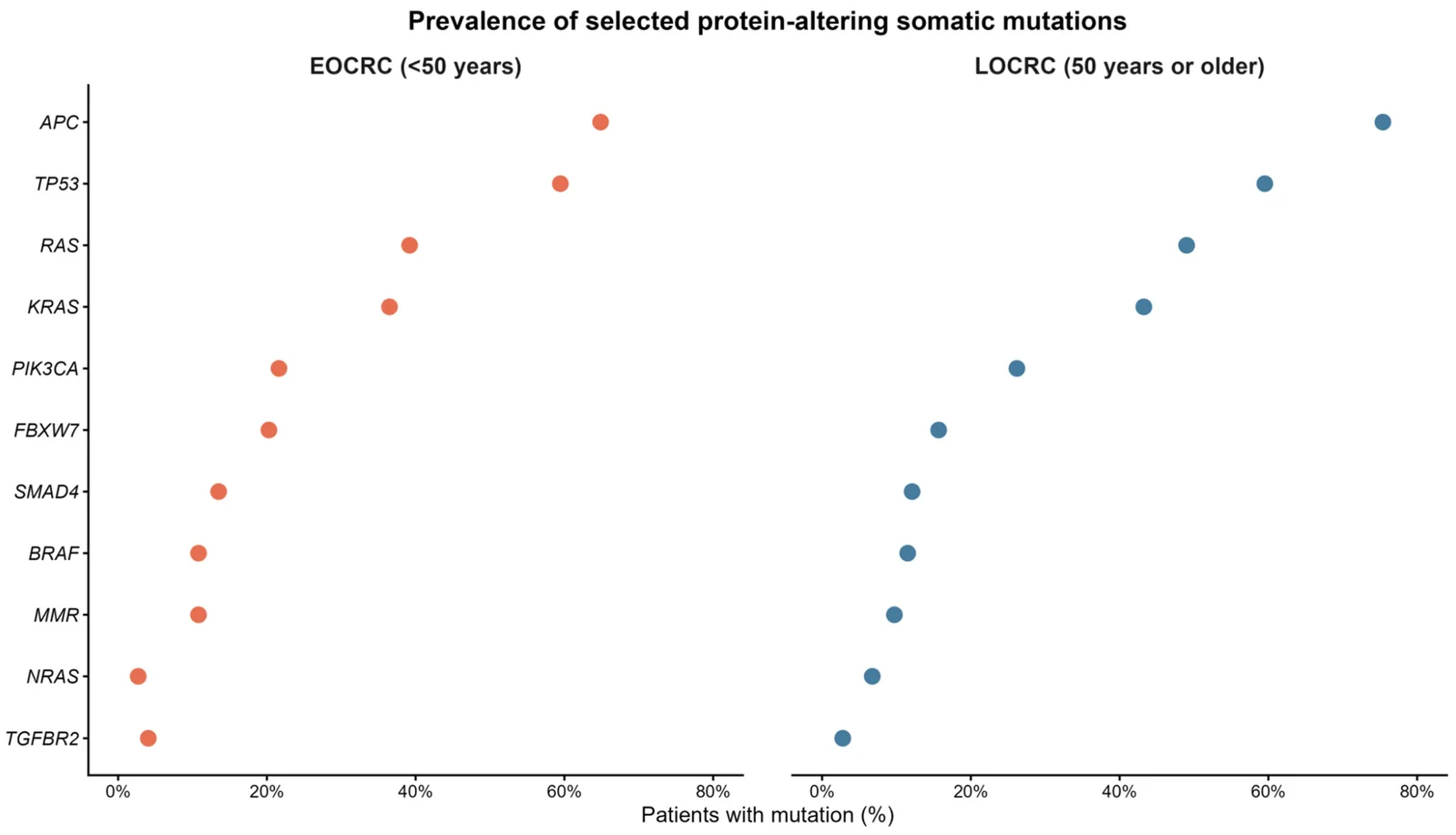
Prevalence of selected protein-altering somatic mutations according to age group. Mutation prevalence was calculated among patients with evaluable somatic mutation data (EOCRC, *n* = 74; LOCRC, *n* = 504). Patients without evaluable mutation data were excluded and were not classified as wild type. Only protein-altering somatic variants were included. EOCRC was defined as colorectal cancer diagnosed before 50 years of age and LOCRC as colorectal cancer diagnosed at 50 years of age or older.

**Table 1:** Clinicopathological characteristics of patients with colorectal cancer according to age at diagnosis in the TCGA-COAD and TCGA-READ cohorts.

Characteristic	LOCRC ≥ 50 Years	EOCRC < 50 Years	*p* Value
Patients, *n*	550	77	-
Age at diagnosis, years, median (IQR)	69.9 (62.4–77.8)	45.2 (40.7–47.4)	-
Year of diagnosis, median (IQR)	2009 (2007–2010)	2010 (2008–2010)	0.007
Sex			0.087
Female	250 (45.5)	43 (55.8)	
Male	300 (54.5)	34 (44.2)	
TCGA project			0.759
TCGA-COAD	402 (73.1)	55 (71.4)	
TCGA-READ	148 (26.9)	22 (28.6)	
Tumor location			0.086
Right colon	229 (43.1)	27 (36.5)	
Left colon	159 (29.9)	26 (35.1)	
Rectosigmoid junction	40 (7.5)	11 (14.9)	
Rectum	103 (19.4)	10 (13.5)	
Histology			0.092
Non-mucinous	487 (88.5)	63 (81.8)	
Mucinous adenocarcinoma	63 (11.5)	14 (18.2)	
AJCC pathological stage			0.021
Stage I	100 (18.8)	9 (12.0)	
Stage II	207 (38.9)	20 (26.7)	
Stage III	151 (28.4)	30 (40.0)	
Stage IV	74 (13.9)	16 (21.3)	
Pathological T category			0.227
T1	17 (3.1)	3 (3.9)	
T2	100 (18.3)	9 (11.7)	
T3	374 (68.3)	52 (67.5)	
T4	57 (10.4)	13 (16.9)	
Pathological N category			0.007
N0	324 (59.2)	31 (40.3)	
N1	126 (23.0)	25 (32.5)	
N2	97 (17.7)	21 (27.3)	
Pathological M category			0.168
M0	412 (84.8)	54 (78.3)	
M1	74 (15.2)	15 (21.7)	
Prior malignancy			0.001
No	479 (87.6)	76 (98.7)	
Yes	68 (12.4)	1 (1.3)	
Synchronous malignancy			0.159
No	519 (94.7)	76 (98.7)	
Yes	29 (5.3)	1 (1.3)	
Residual disease			0.300
R0	401 (90.7)	55 (94.8)	
R1/R2	41 (9.3)	3 (5.2)	
Vital status			0.079
Alive	431 (78.4)	67 (87.0)	
Dead	119 (21.6)	10 (13.0)	

Data are presented as *n* (%) unless otherwise indicated. Percentages were calculated among patients with available data for each variable; therefore, denominators may vary because of missing or unknown information. AJCC pathological stage and T, N, and M subcategories were consolidated into Stages I–IV, T1–T4, N0–N2, and M0–M1, respectively. Pearson’s chi-square or Fisher’s exact test, as appropriate, was used for categorical comparisons, and the Wilcoxon rank-sum test was used for year of diagnosis. Reported *p* values for variables with more than two categories represent overall comparisons. No *p* value was calculated for age because age defined the study groups.

**Table 2:** Somatic mutation profiles according to age group in the TCGA-COAD and TCGA-READ cohorts.

Mutation	LOCRC >= 50 Years, *n*/N (%)	EOCRC < 50 Years, *n*/N (%)	OR (95% CI)	*p* Value	FDR q
*APC*	380/504 (75.4)	48/74 (64.9)	0.60 (0.35–1.06)	0.064	0.691
*TP53*	300/504 (59.5)	44/74 (59.5)	1.00 (0.59–1.70)	1.000	1.000
*KRAS*	218/504 (43.3)	27/74 (36.5)	0.75 (0.44–1.28)	0.314	0.691
*NRAS*	34/504 (6.7)	2/74 (2.7)	0.38 (0.04–1.56)	0.298	0.691
*RAS*	247/504 (49.0)	29/74 (39.2)	0.67 (0.39–1.13)	0.135	0.691
*BRAF*	58/504 (11.5)	8/74 (10.8)	0.93 (0.37–2.08)	1.000	1.000
*PIK3CA*	132/504 (26.2)	16/74 (21.6)	0.78 (0.40–1.43)	0.476	0.749
*SMAD4*	61/504 (12.1)	10/74 (13.5)	1.13 (0.49–2.38)	0.706	0.971
*FBXW7*	79/504 (15.7)	15/74 (20.3)	1.37 (0.68–2.59)	0.314	0.691
*TGFBR2*	14/504 (2.8)	3/74 (4.1)	1.48 (0.27–5.48)	0.468	0.749
MMR-gene mutation	49/504 (9.7)	8/74 (10.8)	1.13 (0.44–2.54)	0.834	1.000

Data are presented as *n*/N (%) among patients with evaluable somatic mutation data (EOCRC, *n* = 74; LOCRC, *n* = 504). Odds ratios compare the odds of mutation in EOCRC with those in LOCRC. Only protein-altering somatic variants were included. *RAS* was defined as a *KRAS* or *NRAS* mutation, and the MMR-gene composite as a somatic mutation in *MLH1*, *MSH2*, *MSH6*, or *PMS2*; the latter does not indicate functional MSI-high/dMMR status. *p* values were calculated using Fisher’s exact test, with Benjamini–Hochberg FDR correction across the 11 comparisons.

**Table 3:** Univariable and multivariable logistic regression analyses of factors associated with early-onset colorectal cancer.

Variable	Univariable OR (95% CI)	*p* Value	Adjusted OR (95% CI)	*p* Value
Diagnosis period		0.003 †		
1998–2005	Reference	-	Reference	-
2006–2009	0.71 (0.34–1.56)	0.370	0.85 (0.39–1.98)	0.695
2010–2013	1.76 (0.89–3.74)	0.119	1.68 (0.81–3.77)	0.179
Sex				
Male	Reference	-	Reference	-
Female	1.52 (0.94–2.47)	0.089	1.41 (0.85–2.38)	0.188
AJCC pathological stage		0.021 †		
Stage I	Reference	-	Reference	-
Stage II	1.07 (0.48–2.56)	0.866	0.89 (0.38–2.17)	0.781
Stage III	2.21 (1.04–5.12)	0.048	2.12 (0.98–5.00)	0.067
Stage IV	2.40 (1.02–5.96)	0.048	2.64 (1.10–6.71)	0.033
Prior malignancy				
No	Reference	-	Reference	-
Yes	0.09 (0.01–0.43)	0.019	0.12 (0.01–0.56)	0.037
*FBXW7* mutation				
Wild type	Reference	-	Reference	-
Mutant	1.37 (0.72–2.48)	0.319	1.46 (0.75–2.73)	0.250

ORs greater than 1 indicate higher odds of EOCRC. The prior-malignancy estimate should be interpreted cautiously because only one EOCRC patient had a documented prior malignancy. † Overall *p* value. Abbreviations: AJCC, American Joint Committee on Cancer; CI, confidence interval; EOCRC, early-onset colorectal cancer; OR, odds ratio.

**Table 4:** Cox proportional hazards regression analyses for overall survival.

Model/Variable	N	Deaths	HR (95% CI)	*p* Value
Model 1—Unadjusted	625	129		
EOCRC vs. LOCRC	625	129	0.60 (0.31–1.14)	0.116
Model 2—Adjusted for sex and diagnosis period	625	129		
EOCRC vs. LOCRC	625	129	0.64 (0.34–1.24)	0.186
Female vs. male	625	129	1.02 (0.72–1.45)	0.911
Diagnosis period 2006–2009 vs. 1998–2005	625	129	0.77 (0.51–1.17)	0.224
Diagnosis period 2010–2013 vs. 1998–2005	625	129	0.52 (0.30–0.87)	0.013
Model 3—Fully adjusted clinicopathological model	584	114		
EOCRC vs. LOCRC	584	114	0.64 (0.33–1.25)	0.194
Female vs. male	584	114	0.98 (0.68–1.43)	0.934
Diagnosis period 2006–2009 vs. 1998–2005	584	114	0.82 (0.52–1.29)	0.383
Diagnosis period 2010–2013 vs. 1998–2005	584	114	0.53 (0.30–0.94)	0.031
Stage II vs. Stage I	584	114	1.77 (0.73–4.27)	0.208
Stage III vs. Stage I	584	114	3.69 (1.55–8.77)	0.003
Stage IV vs. Stage I	584	114	10.09 (4.25–23.93)	<0.001
Left colon vs. right colon	584	114	0.52 (0.33–0.83)	0.006
Rectosigmoid vs. right colon	584	114	0.62 (0.29–1.32)	0.211
Rectum vs. right colon	584	114	0.55 (0.31–0.97)	0.040
Mucinous vs. non-mucinous histology	584	114	1.42 (0.84–2.40)	0.186

HRs and 95% CIs are presented; LOCRC was the reference age group. Model 3 used complete cases for all included clinicopathological covariates (*n* = 584). The proportional hazards assumption was satisfied globally for the fully adjusted model (*p* = 0.091).

**Table 5:** Age-specific associations between somatic mutations and overall survival.

Mutation	LOCRC Mutant *n*/Deaths	LOCRC HR (95% CI)	EOCRC Mutant *n*/Deaths	EOCRC HR (95% CI)	*p* for Interaction	FDR q
*APC*	380/89	0.89 (0.57–1.38)	48/5	0.38 (0.11–1.32)	0.206	0.697
*TP53*	300/74	1.22 (0.83–1.79)	44/5	0.58 (0.17–2.00)	0.261	0.697
*RAS*	247/59	0.94 (0.65–1.36)	29/3	0.85 (0.22–3.30)	0.887	0.887
*BRAF*	58/13	1.01 (0.57–1.80)	8/2	4.37 (0.92–20.74)	0.084	0.670
*PIK3CA*	132/29	0.90 (0.59–1.38)	16/2	1.08 (0.23–5.08)	0.831	0.887
*SMAD4*	61/20	1.47 (0.90–2.38)	10/3	2.73 (0.71–10.58)	0.397	0.793
*FBXW7*	79/16	0.99 (0.58–1.67)	15/2	0.78 (0.17–3.71)	0.784	0.887
MMR-gene mutation	49/13	1.07 (0.60–1.91)	8/1	1.27 (0.16–10.03)	0.877	0.887

Analyses included 577 patients with evaluable mutation and overall-survival data (74 EOCRC and 503 LOCRC; 125 deaths). HRs compare mutant with wild-type patients within each age group. Interaction *p* values were derived from age group-by-mutation Cox models and adjusted across eight tests using the Benjamini–Hochberg method. *RAS* denotes *KRAS* or *NRAS* mutation; the MMR-gene composite denotes a somatic protein-altering mutation in *MLH1*, *MSH2*, *MSH6*, or *PMS2* and does not represent functional MSI-H/dMMR.

## Data Availability

The data analyzed in this study are publicly available through the National Cancer Institute Genomic Data Commons and were derived from the TCGA Colon Adenocarcinoma and TCGA Rectum Adenocarcinoma projects. No new primary data were generated. The complete R script used for cohort construction, mutation processing, MSI sensitivity analyses, statistical analyses, and graphical analyses is provided as Supplementary File S1.

## Supplementary Materials

The following supporting information can be downloaded at: https://www.mdpi.com/article/10.3390/genes17101193/s1, Table S1: Individual mismatch repair gene mutation frequencies according to age group; Table S2: Multivariable logistic regression analysis of pathological nodal status as a sensitivity model for factors associated with early-onset colorectal cancer; Table S3: Expanded multivariable logistic regression model of clinicopathological and genomic factors associated with early-onset colorectal cancer; Table S4: Fully adjusted age-specific association of BRAF mutation with overall survival; Table S5: Sensitivity analysis excluding patients with zero-day survival time; Table S6: Sensitivity analysis using an early-onset colorectal cancer cutoff of <45 years; Table S7: COAD-only sensitivity analysis for overall survival; Table S8: Availability of somatic mutation data according to age group; Table S9: Variant-level distribution of KRAS, BRAF, and PIK3CA protein-altering alterations; Table S10: Microsatellite instability status according to age group; Table S11: MSI-adjusted associations between protein-altering somatic mutations and EOCRC; Table S12: Mutation associations restricted to microsatellite-stable tumors; Table S13: Patient and death counts for exploratory age-by-mutation survival analyses; Table S14: Sensitivity analysis excluding prior malignancy from the EOCRC model. Supplementary File S1: R analysis script.

## Abbreviations

The following abbreviations are used in this manuscript:

AJCC	American Joint Committee on Cancer
CI	Confidence interval
COAD	Colon adenocarcinoma
CRC	Colorectal cancer
dMMR	Deficient mismatch repair
EOCRC	Early-onset colorectal cancer
FDR	False discovery rate
GDC	Genomic Data Commons
HR	Hazard ratio
IQR	Interquartile range
LOCRC	Late-onset colorectal cancer
MMR	Mismatch repair
MSI-H	Microsatellite instability-high
MSS	Microsatellite stable
NCI	National Cancer Institute
OR	Odds ratio
OS	Overall survival
PH	Proportional hazards
READ	Rectum adenocarcinoma
STROBE	Strengthening the Reporting of Observational Studies in Epidemiology
TCGA	The Cancer Genome Atlas
WES	Whole-exome sequencing
