# Supplementary material for: Age-Related Patterns of Selected Protein-Altering Somatic Mutations and Overall Survival in Colorectal Cancer: A Retrospective Comparative Cohort Study
Source: Genes (Basel). 2026 Sep 26;17(10):1193. doi: 10.3390/genes17101193 (PMC13704130; doi:10.3390/genes17101193)
Supplement: Supplementary file 1 [file genes-17-01193-s001.zip › genes-4542343_supplementary.pdf]

Supplementary Materials

Supplementary Table S1. Individual mismatch repair gene mutation frequencies according to age group

Gene	LOCRC n/N (%)	EOCRC n/N (%)	OR (95% CI)	P value	FDR q value
**MLH1**	14/504 (2.8)	4/74 (5.4)	2.00 (0.47–6.60)	0.270	0.540
**MSH2**	14/504 (2.8)	6/74 (8.1)	3.08 (0.94–8.89)	0.032	0.128
**MSH6**	25/504 (5.0)	4/74 (5.4)	1.09 (0.27–3.31)	0.779	0.779
**PMS2**	11/504 (2.2)	3/74 (4.1)	1.89 (0.33–7.40)	0.405	0.540

Footnote: Data are presented as n/N (%) among patients with evaluable somatic mutation data (EOCRC, n = 74; LOCRC, n = 504). Odds ratios compare EOCRC with LOCRC. Fisher exact P values were adjusted across the four MMR genes using the Benjamini–Hochberg method.

Supplementary Table S2. Multivariable logistic regression analysis of pathological nodal status as a sensitivity model for factors associated with early-onset colorectal cancer

Variable	Adjusted OR	95% CI	P value
Diagnosis period			
1998-2005	Reference	-	-
**2006–2009**	0.91	0.42–2.11	0.819
**2010–2013**	1.82	0.89–4.07	0.120
Sex			
Male	Reference	-	-
**Female**	1.48	0.89–2.48	0.131
Pathological N category			
N0	Reference	-	-
**N1**	2.22	1.22–4.01	0.009
**N2**	2.74	1.45–5.14	0.002
Prior malignancy			
No	Reference	-	-
**Yes**	0.12	0.01–0.58	0.040
FBXW7 mutation			
Wild type	Reference	-	-
**Mutant**	1.45	0.74–2.70	0.256

Model characteristics: n = 572; EOCRC cases = 74; LOCRC cases = 498; LR chi-square(7) = 33.49; P < 0.001.

Footnote: This sensitivity model replaced AJCC pathological stage with pathological nodal category to assess whether the association between more advanced nodal disease and EOCRC remained after adjustment. Odds ratios greater than 1 indicate higher odds of EOCRC. The model was adjusted for diagnosis period, sex, pathological N category, prior malignancy, and FBXW7 mutation status. N1 and N2 included their respective pathological substages. NX and missing pathological N values were excluded. Only non-synonymous/protein-altering FBXW7 somatic variants were classified as mutations. Prior malignancy should be interpreted cautiously because only one EOCRC patient had a documented prior malignancy.

Supplementary Table S3. Expanded multivariable logistic regression model of clinicopathological and genomic factors associated with early-onset colorectal cancer

Variable	Adjusted OR	95% CI	P value
Diagnosis period			
1998-2005	Reference	-	-
**2006–2009**	0.79	0.35–1.85	0.564
**2010–2013**	1.52	0.72–3.46	0.291
Sex			
Male	Reference	-	-
**Female**	1.44	0.85–2.46	0.178
Tumor location			
Right colon	Reference	-	-
**Left colon**	1.63	0.86–3.10	0.137
**Rectosigmoid junction**	2.12	0.85–5.01	0.094
**Rectum**	1.08	0.46–2.40	0.846
Histology			
Non-mucinous	Reference	-	-
**Mucinous adenocarcinoma**	1.83	0.85–3.73	0.106
AJCC pathological stage			
Stage I	Reference	-	-
**Stage II**	0.88	0.38–2.18	0.772
**Stage III**	1.88	0.86–4.47	0.131
**Stage IV**	2.35	0.96–6.08	0.067
Prior malignancy			
No	Reference	-	-
**Yes**	0.13	0.01–0.61	0.044
FBXW7 mutation			
Wild type	Reference	-	-
**Mutant**	1.53	0.76–2.96	0.216

Model characteristics: n = 534; EOCRC cases = 69; LOCRC cases = 465; LR chi-square(12) = 34.68; $P < 0.001$.

Footnote: The expanded model additionally included tumor location and histological subtype to assess whether associations observed in the primary model were robust to broader clinicopathological adjustment. Odds ratios greater than 1 indicate higher odds of EOCRC. The model included diagnosis period, sex, tumor location, mucinous histology, AJCC pathological stage, prior malignancy, and FBXW7 mutation status. Only non-synonymous/protein-altering FBXW7 somatic variants were classified as mutations. Complete-case analysis reduced the sample size to 534; this model was therefore considered supplementary rather than primary. Prior malignancy estimates should be interpreted cautiously because only one EOCRC patient had a documented prior malignancy.

Supplementary Table S4. Fully adjusted age-specific association of BRAF mutation with overall survival

Effect	aHR (95% CI)	P value
**EOCRC vs LOCRC among BRAF WT**	0.54 (0.25–1.15)	0.112
**BRAF effect in LOCRC**	0.98 (0.50–1.92)	0.948
**BRAF effect in EOCRC**	3.91 (0.77–19.84)	0.099
**Age × BRAF interaction**	4.00 (0.71–22.45)	0.115

Model: n = 536, deaths = 110; adjusted for sex, AJCC pathological stage, and mucinous histology, with diagnosis period and tumor location included as stratification factors.

Analysis population: n = 536, deaths = 110. The global proportional-hazards test was not significant (P = 0.170).

Supplementary Table S5. Sensitivity analysis excluding patients with zero-day survival time

Variable	aHR (95% CI)	P value
**EOCRC vs LOCRC**	0.68 (0.35–1.34)	0.267
**Female vs male**	0.96 (0.65–1.41)	0.832
**2006–2009 vs 1998–2005**	0.81 (0.50–1.30)	0.379
**2010–2013 vs 1998–2005**	0.58 (0.32–1.06)	0.076
**Stage II vs Stage I**	1.90 (0.73–4.98)	0.191
**Stage III vs Stage I**	4.07 (1.59–10.44)	0.004
**Stage IV vs Stage I**	12.14 (4.76–30.94)	<0.001
**Left colon vs right colon**	0.55 (0.34–0.87)	0.012
**Rectosigmoid vs right colon**	0.63 (0.29–1.37)	0.244
**Rectum vs right colon**	0.60 (0.34–1.07)	0.083
**Mucinous vs non-mucinous**	1.29 (0.73–2.27)	0.376

n = 555; deaths = 107. Global PH test: P = 0.177.

Supplementary Table S6. Sensitivity analysis using an early-onset colorectal cancer cutoff of <45 years

Analysis	HR / aHR (95% CI)	P value
**Unadjusted EOCRC <45 vs CRC ≥45**	0.94 (0.44–2.02)	0.877
**Fully adjusted EOCRC <45 vs CRC ≥45**	1.08 (0.49–2.38)	0.846

Fully adjusted for sex, diagnosis period, AJCC pathological stage, tumor location, and mucinous histology.

Analysis population: unadjusted n = 625, 129 deaths; fully adjusted n = 584, 114 deaths.

Supplementary Table S7. COAD-only sensitivity analysis for overall survival

Variable	aHR (95% CI)	P value
**EOCRC vs LOCRC**	0.79 (0.38–1.61)	0.515
**Female vs male**	0.89 (0.58–1.35)	0.575
**2006–2009 vs 1998–2005**	0.90 (0.54–1.51)	0.690
**2010–2013 vs 1998–2005**	0.49 (0.25–0.95)	0.034
**Stage II vs Stage I**	2.22 (0.77–6.40)	0.140
**Stage III vs Stage I**	4.29 (1.50–12.24)	0.007
**Stage IV vs Stage I**	11.57 (4.05–33.11)	<0.001
**Left colon vs right colon**	0.53 (0.34–0.84)	0.007
**Mucinous vs non-mucinous**	1.43 (0.83–2.47)	0.194

COAD-only analysis; tumor site was restricted to right and left colon. The rectosigmoid category was excluded because it included one patient and no deaths. Analysis population: n = 423, deaths = 90.

Supplementary Table S8. Availability of somatic mutation data according to age group

Age group	Mutation evaluable, n	Mutation unavailable, n	Clinical cohort, n	Evaluable (%)
**EOCRC (<50 years)**	74	3	77	96.1
**LOCRC (≥50 years)**	504	46	550	91.6
**Total**	578	49	627	92.2

Footnote: Mutation-unavailable patients were not classified as wild type and were excluded from mutation-based analyses. Mutation data were evaluable for 74/77 EOCRC patients and 504/550 LOCRC patients.

Supplementary Table S9. Variant-level distribution of KRAS, BRAF, and PIK3CA protein-altering alterations

Gene	Variant group	LOCRC patients, n	EOCRC patients, n
KRAS	Codon 117	5	1
KRAS	Codon 12	141	19
KRAS	Codon 13	40	3
KRAS	Codon 146	16	2
KRAS	Codon 61	11	1
KRAS	Other KRAS variant	6	2
BRAF	Non-V600E BRAF variant	15	5
BRAF	V600E hotspot-equivalent annotation	47	3
PIK3CA	Helical-domain hotspot region	59	5
PIK3CA	Kinase-domain hotspot region	34	3
PIK3CA	Other PIK3CA variant	52	10

Footnote: Counts are descriptive and may exceed the number of gene-mutant patients because a patient could harbor more than one qualifying variant. The GDC transcript annotation labels the canonical BRAF hotspot-equivalent event as p.V640E; therefore, hotspot-equivalent terminology is used pending transcript-level confirmation.

Supplementary Table S10. Microsatellite instability status according to age group

Age group	MSI, n/N (%)	MSS, n/N (%)
EOCRC (<50 years)	9/74 (12.2)	65/74 (87.8)
LOCRC (>=50 years)	71/514 (13.8)	443/514 (86.2)

Footnote: MSI status was obtained from GDC whole-exome sequencing metadata generated using MSIsensor2.

Five patients with discordant MSI/MSS records were excluded. Fisher exact P = 0.856.

Supplementary Table S11. MSI-adjusted associations between protein-altering somatic mutations and EOCRC

Mutation	N	MSI-adjusted OR (95% CI)	P value	FDR q
**APC**	572	0.55 (0.33–0.96)	0.032	0.356
**TP53**	572	0.95 (0.57–1.59)	0.833	0.916
**KRAS**	572	0.74 (0.44–1.22)	0.245	0.674
**NRAS**	572	0.39 (0.06–1.34)	0.207	0.674
**RAS**	572	0.65 (0.39–1.08)	0.099	0.545
**BRAF**	572	1.00 (0.37–2.43)	1.000	1.000
**PIK3CA**	572	0.74 (0.39–1.32)	0.329	0.708
**SMAD4**	572	1.17 (0.54–2.31)	0.674	0.824
**FBXW7**	572	1.33 (0.67–2.49)	0.386	0.708
**TGFBR2**	572	1.59 (0.35–5.25)	0.488	0.766
**MMR-gene mutation**	572	1.30 (0.50–3.05)	0.571	0.785

Footnote: Logistic regression models compared EOCRC with LOCRC and adjusted for functional MSI status. Five patients with discordant MSI/MSS records were excluded. Each model included 572 patients with complete MSI and mutation data (73 EOCRC and 499 LOCRC). FDR was controlled across 11 prespecified comparisons using the Benjamini–Hochberg method.

Supplementary Table S12. Mutation associations restricted to microsatellite-stable tumors

Mutation	LOCRC n/N (%)	EOCRC n/N (%)	OR (95% CI)	P value	FDR q
**APC**	347/431 (80.5)	42/64 (65.6)	0.46 (0.25–0.86)	0.009	0.072
**TP53**	276/431 (64.0)	42/64 (65.6)	1.07 (0.60–1.96)	0.889	1.000
**KRAS**	193/431 (44.8)	20/64 (31.2)	0.56 (0.30–1.01)	0.043	0.119
**NRAS**	31/431 (7.2)	2/64 (3.1)	0.42 (0.05–1.71)	0.291	0.533
**RAS**	221/431 (51.3)	22/64 (34.4)	0.50 (0.27–0.89)	0.015	0.072
**BRAF**	16/431 (3.7)	7/64 (10.9)	3.17 (1.06–8.59)	0.020	0.072
**PIK3CA**	103/431 (23.9)	11/64 (17.2)	0.66 (0.30–1.34)	0.268	0.533
**SMAD4**	49/431 (11.4)	9/64 (14.1)	1.28 (0.52–2.82)	0.533	0.733
**FBXW7**	53/431 (12.3)	10/64 (15.6)	1.32 (0.56–2.83)	0.426	0.670
**TGFBR2**	7/431 (1.6)	1/64 (1.6)	0.96 (0.02–7.69)	1.000	1.000
**MMR-gene mutation**	19/431 (4.4)	3/64 (4.7)	1.07 (0.20–3.78)	1.000	1.000

Footnote: The analysis was restricted to mutation-evaluable MSS tumors (EOCRC, n = 64; LOCRC, n = 431). Odds ratios compare EOCRC with LOCRC. No comparison remained significant after Benjamini–Hochberg FDR correction.

Supplementary Table S13. Patient and death counts for exploratory age-by-mutation survival analyses

Mutation	Age group	Mutation status	Patients, n	Deaths, n
**APC**	EOCRC	Mutant	48	5
**APC**	EOCRC	Wild type	26	5
**APC**	LOCRC	Mutant	380	89
**APC**	LOCRC	Wild type	123	26
**TP53**	EOCRC	Mutant	44	5
**TP53**	EOCRC	Wild type	30	5
**TP53**	LOCRC	Mutant	300	74
**TP53**	LOCRC	Wild type	203	41
**RAS**	EOCRC	Mutant	29	3
**RAS**	EOCRC	Wild type	45	7
**RAS**	LOCRC	Mutant	247	59
**RAS**	LOCRC	Wild type	256	56
**BRAF**	EOCRC	Mutant	8	2
**BRAF**	EOCRC	Wild type	66	8
**BRAF**	LOCRC	Mutant	58	13
**BRAF**	LOCRC	Wild type	445	102
**PIK3CA**	EOCRC	Mutant	16	2
**PIK3CA**	EOCRC	Wild type	58	8
**PIK3CA**	LOCRC	Mutant	132	29
**PIK3CA**	LOCRC	Wild type	371	86
**SMAD4**	EOCRC	Mutant	10	3
**SMAD4**	EOCRC	Wild type	64	7
**SMAD4**	LOCRC	Mutant	61	20
**SMAD4**	LOCRC	Wild type	442	95
**FBXW7**	EOCRC	Mutant	15	2
**FBXW7**	EOCRC	Wild type	59	8
**FBXW7**	LOCRC	Mutant	79	16
**FBXW7**	LOCRC	Wild type	424	99
**MMR-gene mutation**	EOCRC	Mutant	8	1
**MMR-gene mutation**	EOCRC	Wild type	66	9
**MMR-gene mutation**	LOCRC	Mutant	49	13
**MMR-gene mutation**	LOCRC	Wild type	454	102

Footnote: Counts correspond to the mutation-evaluable overall-survival cohort used for the unadjusted interaction models (n = 577; 74 EOCRC and 503 LOCRC; 125 deaths).

Supplementary Table S14. Sensitivity analysis excluding prior malignancy from the EOCRC model

Variable	Adjusted OR (95% CI)	P value
**Diagnosis period 2006–2009 vs 1998–2005**	0.81 (0.37–1.87)	0.604
**Diagnosis period 2010–2013 vs 1998–2005**	1.86 (0.91–4.16)	0.105
**Female vs male**	1.47 (0.88–2.47)	0.139
**Stage II vs Stage I**	0.88 (0.38–2.15)	0.764
**Stage III vs Stage I**	2.09 (0.97–4.90)	0.072
**Stage IV vs Stage I**	2.49 (1.04–6.29)	0.044
**FBXW7 mutant vs wild type**	1.47 (0.75–2.73)	0.240

Footnote: Prior malignancy was omitted because only one EOCRC patient had a prior malignancy. The model otherwise included diagnosis period, sex, AJCC pathological stage, and FBXW7 mutation status. The complete-case model included 558 patients (72 EOCRC and 486 LOCRC).
